# The association between organizational behavior factors and health-related quality of life among college teachers: a cross-sectional study

**DOI:** 10.1186/s12955-015-0287-4

**Published:** 2015-06-20

**Authors:** Chuan Liu, Shu Wang, Xue Shen, Mengyao Li, Lie Wang

**Affiliations:** Department of Social Medicine, School of public health, China Medical University, No.92 North Second Road, Heping District, Shenyang 110001 China

**Keywords:** College teacher, Health-related quality of life, Psychological capital, Group identification, Organizational justice, Perceived organizational support, Psychological empowerment

## Abstract

**Background:**

College teachers in China are confronted with a lot of pressure from teaching, researching and living. They are suffering from impaired physical and mental health. The purpose of this study was to investigate the relationships between organizational behavior factors and college teachers’ health related quality of life (HRQOL), and to confirm whether they are positive resources for improving teachers’ HRQOL.

**Methods:**

A cross-sectional survey was conducted in Shenyang, China, from January to April 2014. Participants were composed of 965 teachers randomly selected from five representative colleges in Shenyang. Self-administrated questionnaires containing the 36-item Short-Form Health Survey (SF-36), the Chinese version Psychological Capital Questionnaire (PCQ), and scales assessing group identification, POS, and psychological empowerment were used to measure HRQOL and organizational behavior variables of college teachers. Hierarchical multiple regression analysis (HMR) was performed to explore the effects of organizational behavior variables on college teachers’ HRQOL.

**Results:**

The mean (SD) scores of physical component summary (PCS) and mental component summary (MCS) among college teachers were 71.43 (14.70) and 65.46 (16.55) respectively in the study population. Hierarchical multiple regression analysis showed that group identification (β = 0.121, *P* < 0.001) and PsyCap (β = 0.336, *P* < 0.001) were significant predictors of PCS, while group identification (β = 0.107, *P* < 0.001), POS (β = 0.124, *P* = 0.003), psychological empowerment (β = 0.093, *P* = 0.017) and PsyCap (β = 0.421, *P* < 0.001) were significant predictors of MCS.

**Conclusion:**

Chinese college teachers experienced relatively low level of HRQOL and their mental quality of life (QOL) were impaired more seriously than physical QOL. Organizational behavior factors (PsyCap, group identification, POS and psychological empowerment) were strong predictors of college teachers’ HRQOL and are positive resources for improving teachers’ HRQOL. The enhancement of college teachers’ PsyCap, group identification, POS and psychological empowerment at work should be incorporated in the strategy of protecting and improving college teachers’ physical and mental QOL.

## Introduction

College teachers play a crucial role in society as they bear the vital tasks of cultivating highly qualified special talents and developing science and culture. Since 1999, Chinese government proposed an expansion of university enrolment to ensure that the output of specialized graduates could meet the needs of the rapid economic development. In China’s academic profession, faculty hiring has been shift from “identity management” to “position management”, and the payment for performance accounts for a bigger proportion of the whole salary [1]. Under this context, college teachers in China are confronted with a lot of pressure from teaching, researching and living [2]. Teacher is considered to be a highly stressful career, and there have been various studies showed that teachers are frequently under high pressure [3–5]. They have a higher prevalence of mental and physical problems like anxiety, hypertension, headaches, psychosomatic disorders and cardiovascular diseases compared with other workers [6–8]. And it has been reported that the quality of life (QOL) among Chinese college teachers was lower than that of the Chinese general population [9, 10].

There have been various studies focusing on the influencing factors of teachers’ QOL. Ge et al. investigated the influences of demographic variables (age, gender, education, and marital status), work-related factors (role overload, job rank, working hours, role boundary and number of SCI papers) and chronic disease on college teachers’ QOL [9]. In another study on the physical activity level and life quality of teachers, a negative meaningful relation among weight and activity level as well as life quality was observed [11]. Earlier finding showed that the more stress the teacher experienced from work overload, organizational climate and staff relations factors, the more impaired was teachers’ QOL [12]. In general, previous researches concerning the influencing factors of teachers’ QOL have been confined to the perspective of individuals, while factors at the organizational level were rarely taken into consideration.

Organizational behavior (OB) is the study of human behavior in organizational settings, of the interface between human behavior and the organization, and of the organization itself [13]. OB is an exciting and complex field of study. The specific concepts and topics that constitute the field can be grouped into three categories: individual, interpersonal, and organizational processes and characteristics [13]. OB provides guidance in understanding, appreciating and managing others in organization, and also provides unique and important opportunities to enhance personal and organizational effectiveness [13]. Organizational behavior variables discussed in this study include psychological capital (PsyCap), group identification, organizational justice, perceived organizational support (POS) and psychological empowerment.

PsyCap is a kind of positive psychological state of an individual in the process of growth and development and is a higher-order core construct drawn from positive organizational behavior [14]. Luthans and his colleagues identified that the positive psychological constructs meeting the inclusion criteria so far included hope, resilience, optimism, and self-efficacy, and represented PsyCap when combined [15]. Self-efficacy represents having the confidence to succeed at challenging tasks; hope is defined as persevering toward goals and, when necessary, redirecting paths to goals in order to succeed; resilience is the positive psychological capacity to sustain and bounce back and even beyond to attain success; optimism is the inclination to make a positive attribution about succeeding now and in the future [16, 17]. Each of these four positive constructs has conceptual independence, but there is a common, underlying link that runs between them and ties them together, that is, a mechanism shared across each of the facets that contribute to a motivational propensity to accomplish tasks and goals [16]. Those high in self-efficacy will be more resilient to adversity, and those high in hope tend to be more confident on specific tasks (self-efficacy) and are quickly able be bounce back (resilience) after temporary hopelessness [16]. PsyCap, as a psychological state that can lead to positive organization behavior, has been reported to be a positive resource for combating occupational stress and turnover [14], depression [18] and burnout [19].

Group identification is defined as member identification with an interacting group and is proposed to have three sources: cognitive (social categorization), affective (interpersonal attraction), and behavioral (interdependence) [20]. The cognitive source taps how social identity and social categorization-aspects of individual cognition and the self-influence group identification. Cognitive process of self-categorization can either facilitate or hinder the emergence of group identification. Depersonalized attraction is an affective consequence of cognitive judgment about joint membership and evokes an intergroup reference frame. The affective source focuses on the contribution of interpersonal attraction (interpersonal level). Group identification develops as a result of affective bonds among group members. If members are attracted to one another, they may prefer to spend more time together as interaction leads to goal attainment. If members interact cooperatively to attain shared goals, then this collection of individuals who feel mutual attraction has become a group of interdependence members. The behavioral source focuses on the group-level construct of cooperative interdependence. Interdependence causes in-group formation, a necessary backdrop for attraction to a group and categorization of oneself as a member. In this way, the three sources work together to promote member identification with an interacting group [20]. According to social identity researchers, group identification could protect against existential anxiety [21], and it has been confirmed that greater group identification is associated with better mental health [22, 23]. Meanwhile, very few are known about the effects of group identification on physical health.

Organizational justice is a construct defining the equality of social interaction at work [24]. Colquitt identified four distinct categories of organizational justice: procedural justice, distributive justice, interpersonal justice and information justice [25]. Distributional justice is allocation of the enterprise’s source that is defined in accordance with the some rules and standards between workers; Procedural justice is a level of the justice in the methods, procedures and politics that are used to determine wage, carrier, working conditions and performance evaluation criteria in working place; Interpersonal Justice is social aspect of the organizational justice and emphasizes outcomes of the investment that made to improve the relationship between persons [26].By reducing secrecy and dishonesty, information justice illustrates the kind of trustworthiness that can increase status judgments and collective esteem [25]. These four components of organizational justice are statistically separated constructs; they seemed to be distinct but have usually been combined because of high intercorrelations [25]. Justice is a basic requirement for the effective functioning of organizations and personal satisfaction of the individuals they employ [27]. A review of evidence on organizational justice and health suggests that organizational justice is related to employees’ health and well-being [28]. Low perceived justice has been shown to be associated with experienced stress reactions and related physiological and behavioral reactions, such as inflammation, sleeping problems, cardiovascular regulation and cognitive impairments [28].

POS is an employee belief concerning the extent to which the organization values their contribution and cares about their well-being [29]. Greater POS is expected to result in greater affective attachment and feelings of obligation to the organization [30]. A meta-analysis of the relationship between POS and job outcomes indicates that POS has a positive effect on job satisfaction, organizational commitment and employee performance, and a negative effect on intention to leave [31]. Employees with higher POS reported lower levels of depression, anxiety, concern for their health [32]. It is also reported that the higher POS, the lower emotional exhaustion, and the better psychological health [33] and a positive relation between POS and physical health is also confirmed [34].

Psychological empowerment is defined as a motivational construct manifested in four cognitions: meaning, competence, self-determination, and impact [35]. Together, these four cognitions reflect an active, rather than a passive, orientation to a work role [35]. Meaning is the value of a work goal or purpose, judged in relation to an individual’s own ideals or standards; competence is an individual’s belief in his or her capability to perform activities with skills; self-determination is an individual’s sense of having choice in initiating and regulating actions; impact is the degree to which an individual can influence strategic, administrative, or operating outcomes at work [35]. The four dimensions are argued to combine additively to create an overall construct of psychological empowerment, and the lack of any single dimension will deflate, though not completely eliminate, the overall degree of felt empowerment [35]. Psychological empowerment was positively related to work satisfaction and higher senses of psychological empowerment was related to higher job effectiveness and lower strain [36]. Moreover, psychological empowerment at work is positively correlated with general mental health, and negatively correlated with burnout and sick leave [37]. It is a possible factor that provides protection against ill health [37].

**Fig. 1:**
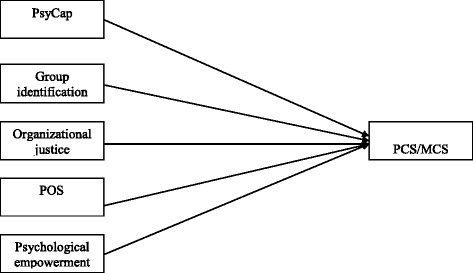
Conceptual map with the hypothesized relations. PsyCap: psychological capital; POS: perceived organizational support; PCS: physical component summary; MCS: mental component summary

To our knowledge, studies conducted to explore the effects of these organizational behavior variables on college teachers’ health related quality of life (HRQOL) are rarely found. And according to the reviewed literature above, these variables probably could be potential resources for enhancing the QOL of teachers. This study aims to investigate the relationships between organizational behavior variables (PsyCap, group identification, organizational justice, POS and psychological empowerment) and teachers’ HRQOL, and to confirm whether they are positive resources for improving teachers’ HRQOL(Conceptual map see Fig. 1), which could provide practical suggestions as to how colleges can improve their teachers’ health, thus improving organizational effectiveness.

## Methods

### Study design and sample

This study incorporated a cross-sectional design and was conducted from January to April 2014. The sample of this research was composed of 965 teachers randomly selected from five representative colleges (Shenyang Ligong University, Shenyang University of Chemical Technology, Shenyang Normal University, Shenyang Institute of Engineering and Shenyang Aerospace University) of Shenyang, the capital city of Liaoning Province located in northeast China. With the help of Shenyang Science-Education-Culture-Health Union, who chose these five representative colleges on the basis of assessing the education scale, academic level and subject category, a self-administered questionnaire was distributed to and gathered from teachers of the five aforementioned colleges. After obtaining the informed consent to carry out the research, a total of 1500 were distributed to teachers and effective responses were obtained from 965 teachers (effective response rate: 64.3 %). The study protocol and informed consent form were in accordance with the standard of the Committee on Human Experimentation of China Medical University and received approval.

### Measures

#### Demographic and work characteristics of teachers

Demographic characteristics of college teachers included gender, age, marital status, educational level, life events and chronic disease. Information of work situation regarding working years (number of years working for the given college), monthly income, job title, weekly working hours, number of SCI /SSCI and turnover intention (the intention to quit the present job) were obtained. “Marital status” was categorized as “single”, “married/ cohabitation” and “divorced /others”. “Educational level” was categorized as “doctor”, “master” and “bachelor”. As for life events, respondents were asked a simple dichotomous question: “Have you experienced any life events (i.e. death, illness, or laid-off of kinsfolk, entering a higher school or getting married of children) during the past year? (Yes /No)”. “Chronic disease” was defined as “yes” if common chronic disease (e.g. hypertension, hyperlipidemia, fatty liver, diabetes, gout, hyperuricemia, coronary heart disease, Chronic Gastritis and lumbodorsalgia) was diagnosed. “Job title” was categorized as “professor”, “associate professor”, “lecturer” and “assistant”. In China, university graduates with master degrees are qualified for the promotion to Lecturer usually after a minimum of 2-year working experiences. Those with doctorate degrees are qualified for the promotion to associate professor usually after a minimum of 2-year working experiences. Associate professor, usually after a minimum of 5-year working experiences, is qualified for the promotion to Professor. “Monthly income (RMB)” was categorized as “<3000”, “3000-4999”, “5000-6999”, and “≥7000”. “Weekly working hours” was categorized as “≤40 h” and “>40 h”. “Turnover intention” was categorized as “never”, “sometimes” and “frequently”.

### Assessment of QOL

The 36-item Short-Form Health Survey (SF-36) was used to assess QOL of college teachers. The SF-36 measures eight different concepts of health: physical functioning (PF), role limitations due to physical problems (RP), bodily pain (BP), general health (GH), vitality (VT), social functioning (SF), role limitations due to emotional problems (RE), and mental health (MH) (e.g., “Have you experienced body pain in the last four weeks?”). These eight dimensions can be aggregated into physical component summary (PCS) and mental component summary (MCS). A score was calculated for each dimension and was transformed to obtain a value ranging from 0 to 100, with higher scores indicating better health [38]. SF-36 is a generic health questionnaire originally developed in the United States [39] and the Mandarin version of SF-36 is a valid and reliable tool for assessing HRQOL [40]. The Cronbach’s alpha for PCS and MCS in this study were 0.898 and 0.806, respectively.

### Assessment of organizational behavior variables

The survey questionnaire contained a number of instruments measuring organizational behavior variables (i.e. psychological capital, group identification, organizational justice, perceived organizational support and psychological empowerment). All the items of the instruments were translated into Chinese. An average score of each scale is calculated to get a composite construct and all items were coded so that a higher score indicated a higher level of the construct being measured.

### Psychological capital

The Chinese version Psychological Capital Questionnaire (PCQ) developed by Luthans et al. [41] was applied to measure PsyCap. The PCQ consists of 24 items each of which is scored on a 6-point Likert scale and 1 represents strongly disagree and 6 represents strongly agree (e.g., “I feel confident in analyzing a long-term problem to find a solution.”). Each of the four dimensions of PsyCap, i.e. self-efficacy, hope, resilience and optimism is measured by six items. The Chinese version of the PCQ has been used in various Chinese studies with satisfactory reliability and validity [18, 19, 42]. In this study, Cronbach’s alpha for self-efficacy, hope, resilience, optimism and the total scale was 0.880, 0.904, 0.816, 0.715 and 0.944respectively.

### Group identification

A 12-item scale based on the tripartite model of group identification developed by Henry et al. was used to measure group identification (e.g., “I would prefer to be in a different group”) [20]. This scale have been demonstrated good reliability and reasonable stability of the factor structure and is composed of three dimensions: cognitive, affective and behavioral, each of which is measured by four items [20]. For each item, the scale ranged from 1(strongly disagree) to 7 (strongly agree). The Cronbach’s alpha for cognitive, affective, behavioral and the total scale was 0.655, 0.716, 0.563 and 0.849 respectively in our study which indicated good reliability in Chinese population.

### Organizational justice

Organizational justice was measured with Colquitt’s 20-item scale, which measures four dimensions of organizational justice: procedural justice, distributive justice, interpersonal justice and information justice (e.g., “Have you been able to express your views and feelings during those procedures?”) [25]. Items of this scale were generated by strictly following the seminal works in the justice literature and it has good reliability and construct validity [25]. Colquitt’s organizational justice scale is a 5-point Likert scale, items ranging from 1(strongly disagree) to 5 (strongly agree). The Cronbach’s alpha of the four dimensions and the total scale was 0.890, 0.926, 0.933, 0.944 and 0.956 respectively in this study, which indicated rather good reliability in Chinese population.

### Perceived organizational support

A 9-item short form of the survey of Perceived Organizational Support developed by Eisenberger et al. [29] was employed to measure POS (e.g., “The organization cares about my general satisfaction at work”). The nine items of this scale are items with the highest factor loading of the 36-version of this questionnaire [43]. Each item had seven response categories ranging from 1 (strongly disagree) to 7 (strongly agree). This shortened version of the POS has been used in various Chinese researches with good reliability and validity [44, 45]. The Cronbach’s alpha was 0.918 in this study.

### Psychological empowerment

Spreitzer’s empowerment scale was used to measure psychological empowerment [35]. It is a 12-item scale measuring four dimensions of psychological empowerment: meaning, competence, self-determination, and impact, and each dimension is measured by three items (e.g., “The work I do is very important to me”). The response scale is 7-point Likert ranging from 1 (strongly disagree) to 7 (strongly agree). This scale has been proved to have good reliability and satisfactory convergent and discriminant validity [35]. For the present study, The Cronbach’s alpha of the four dimensions and the total scale was 0.939, 0.944, 0.927, 0.939 and 0.946 respectively, which indicated rather good reliability in Chinese population.

### Statistical analysis

The data acquired in this study were analyzed by SPSS v13.0 statistical program for windows. Differences of QOL in categorical variables were examined by *t*-test and one-way ANOVA. Pearson’s correlation was performed to test the correlation among continuous variables. All statistical tests were two-sided (α = 0.05). With demographic and work characteristics and organizational behavior factors as independent variables, Hierarchical Multiple Regression analysis was conducted to test the incremental variance by independent variables (i.e. PCS and MCS scores). We used tolerance and variance inflation factor to check for multicollinearity, and no multicollinearity was found in this study. In the first step of the hierarchical linear regression, demographic and work characteristics were entered into the regression model. In the second step, group identification, organizational justice, POS, psychological empowerment and PsyCap were added.

## Results

### Participant characteristics

Participant characteristics were presented in Table 1. Effective responses were obtained from 965 college teachers. The mean (S.D.) age of participants was 37.53 (6.70) years, ranging from 20 to 65 years. There were 422 (43.7 %) males and 543 (56.3 %) females. With respect to marital status, 87.0 % of the teachers were married/cohabitation and 11.0 % were single and 2.0 % were divorced/others. 28.2 % teachers had doctor degree, 59.5 % had master degree and 12.3 % had bachelor degree. Working years of teachers ranged from 1 to 40 years. The majority of teachers (59.9 %) had the monthly income (RMB) of 3000–4999, 27.9 % had 5000–6999, 6.2 % had ≥7000 and 6.0 % had <3000. In regard to job title, 6.9 % of the teachers were professors, 32.6 % were associate professors, 52.7 % were lecturers and 7.7 % were assistants. 20.3 % worked more than 40 hours a week while only 15.5 % have published SCI/SSCI in the last 3 years. Meanwhile, 36.7 % have turnover intention sometimes and 4.4 % frequently have. Moreover, 53.3 % of teachers experienced life events and 42.7 % had chronic diseases.

**Table 1:** Demographic and work characteristics of college teachers, means and standard deviations of PCS and MCS

Variable	N (%)	PCS (Mean ± SD)	MCS (Mean ± SD)
Total Gender	965(100)	71.43 ± 14.70	65.46 ± 16.55
Male	422(43.7)	71.55 ± 15.65	65.25 ± 16.50
Female	543(56.3)	71.35 ± 13.93	65.63 ± 16.60
Age			
≤30	113(11.7)	76.11 ± 12.03**	68.97 ± 16.85*
31-40	591(61.2)	70.93 ± 14.89	64.77 ± 16.70
41-50	213(22.1)	70.57 ± 15.51	65.07 ± 16.47
>50	48(5.0)	70.53 ± 12.59	67.48 ± 13.25
Marital status			
Single	106(11.0)	72.34 ± 13.84	63.98 ± 19.27
Married/cohabitation	840(87.0)	71.24 ± 14.84	65.51 ± 16.19
Divorced/Others	19(2.0)	75.16 ± 13.21	71.57 ± 15.51
Educational level			
Doctor	272(28.2)	73.03 ± 15.42*	66.25 ± 16.82
master	574(59.5)	70.44 ± 14.30*	64.46 ± 16.63*
bachelor	119(12.3)	72.61 ± 14.65	68.46 ± 15.17*
Monthly income(RMB)			
<3000	58(6.0)	74.47 ± 13.44	66.11 ± 17.34
3000-4999	578(59.9)	71.71 ± 14.12	65.99 ± 16.24
5000-6999	269(27.9)	70.05 ± 15.99	63.68 ± 17.57
≥7000	60(6.2)	72.08 ± 15.09	67.71 ± 13.49
Job title			
Professor	67(6.9)	70.82 ± 15.75	66.99 ± 14.77
Associate professor	315(32.6)	70.95 ± 15.54	64.34 ± 17.49*
Lecturer	509(52.7)	71.15 ± 14.13	65.47 ± 15.94
Assistant	74(7.7)	76.03 ± 13.36*	68.81 ± 17.84*
Weekly working hours			
≤40	769(79.7)	72.15 ± 14.49**	66.56 ± 16.26***
>40	196(20.3)	68.64 ± 15.23	61.15 ± 17.03
Number of SCI/SSCI (last 3 years)			
0	815(84.5)	71.17 ± 14.73	65.50 ± 16.58
1 ~ 3	111(11.5)	72.94 ± 14.75	65.73 ± 17.40
>3	39(4.0)	72.73 ± 13.89	63.99 ± 13.64
Turnover intention			
Never	532(58.8)	74.10 ± 13.88***	68.67 ± 15.93***
Sometimes	332(36.7)	67.13 ± 15.13	60.98 ± 15.74
Frequently	40(4.4)	68.22 ± 15.48	57.88 ± 20.67
Life events			
No	434(46.7)	72.74 ± 14.31**	67.29 ± 16.03**
Yes	496(53.3)	69.89 ± 14.98	63.56 ± 16.70
Chronic diseases			
No	553(57.3)	74.84 ± 13.64***	68.73 ± 16.00***
Yes	412(42.7)	66.86 ± 14.85	61.08 ± 16.28

*PCS* physical component summary; *MCS* mental component summary; *SD* standard deviation

*p < 0.05, **p < 0.01, ***p < 0.001

### Description of college teachers’ QOL

In this study, the mean and standard deviation of PCS and MCS among college teachers were 71.43 (14.70) and 65.46 (16.55) respectively, and mental QOL was significantly lower than physical QOL (*p* = 0.026). Mean scores of PCS and MCS based on demographic and work characteristics of college teachers were also shown in Table 1. Teachers who were ≤ 30 years old had significantly higher PCS than all the other three age subgroups (F = 4.403, *p* = 0.004), and they also had significantly higher MCS than 31–40 (*p* = 0.013) and 41–50 (*p* = 0.043) subgroups. Teachers with doctor degree had significantly higher PCS than those with master degree (*p* = 0.017), while those with bachelor degree had significantly higher MCS than that of those with master degree (*p* = 0.017). As for job title, assistants had significantly higher PCS than other three job title subgroups (F = 2.639, *p* = 0.048), and had significant higher MCS than associate professors (*p* = 0.037). Teachers working more than 40 h per week, experiencing life events or diagnosed with chronic diseases tended to have lower level of PCS and MCS. What’s more, teachers who never had turnover intention had significant higher PCS and MCS scores than those who sometimes or significantly had turnover intention (PCS: F = 24.787, p < 0.001; MCS: F = 27.840, *P* < 0.001). No significant differences of PCS and MCS scores across each of all the other characteristic subgroups were found.

### Correlation between study variables and predictors of college teachers’ QOL

Results of Pearson correlation were provided in Table 2. All the organizational behavior variables were significantly positively correlated with PCS and MCS. And group identification, organizational justice, POS, psychological empowerment and PsyCap were significantly positively correlated with each other. Chronic disease was significantly negatively correlated with organizational justice, POS, psychological empowerment and PsyCap, and negatively correlated with PCS and MCS. The results of hierarchical multiple regression models of college teachers’ QOL were presented in Table 3 and Table 4. In the final regression model, 33.3 % variance of PCS and 45.5 % variance of MCS were explained. In the multiple regression models, group identification (β = 0.121, *P* < 0.001) and PsyCap (β = 0.335, *P* < 0.001) were significant predictors of PCS, while group identification (β = 0.107, *P* < 0.001), POS (β = 0.124, *P* = 0.003), psychological empowerment (β = 0.093, *P* = 0.017) and PsyCap (β = 0.420, *P* < 0.001) were significant predictors of MCS. The standardized coefficients of organizational justice, POS, and psychological empowerment were not significant in the regression analysis for PCS, and the standardized coefficient of organizational justice was not significant for MCS. In the regression model of PCS, after adding OB variables, the coefficients of “working years” changed from “-0.050*” to “-0.025”, the coefficients of “job title (Lecturer vs. Assistant)” changed from “-0.140*” to “-0.066”, and the coefficients of “chronic disease” changed from “-0.238***” to “-0.180***” (Table 3). Similar change of the coefficients of some variables can also be found in the regression model of MCS (Table 4). These indicate that, under multi-factor circumstances, the effects of these variables on HRQOL were reduced or, more accurately, mediated by OB factors. Organizational behavior variables (PsyCap, group identification, organizational justice, POS and psychological empowerment) together contribute 21.9 % and 34.7 % to the variance of PCS and MCS respectively.

**Table 2:** Means, standard deviations, and correlation among study variables

	Mean	SD	1	2	3	4	5	6	7
1.group identification	5.31	1.03							
2.organizational justice	3.34	0.77	0.428**						
3.POS	4.55	1.17	0.432**	0.675**					
4.psychological empowerment	4.73	1.16	0.404**	0.554**	0.678**				
5.PsyCap	4.17	0.75	0.452**	0.525**	0.671**	0.693**			
6.PCS	71.43	14.7	0.340**	0.359**	0.436**	0.435**	0.509**		
7.MCS	65.46	16.55	0.391**	0.413**	0.533**	0.526**	0.625**	0.684**	
8.Chronic diseases	1.43	0.5	−0.059	−0.181**	−0.190**	−0.163**	−0.171**	−0.269**	−0.229**

*POS* perceived organizational support; *PsyCap* psychological capital; *PCS* physical component summary; *MCS* mental component summary

*p < 0.05, **p < 0.01, ***p < 0.001

**Table 3:** Hierarchical multiple regression predicting the PCS scores

Variable		PCS	
		Step1(β)	Step2(β)
Demographic and work characteristics			
Age		−0.003	−0.003
Gender	(Man vs. Women)	0.010	0.017
Marital status			
	(Single vs. Married/cohabitation)	−0.023	−0.028
	(Divorced/others vs. Married/cohabitation)	0.042	0.020
Educational level			
	(Doctor vs. bachelor)	0.006	0.007
	(master vs. bachelor)	−0.092	−0.050
Working years		−0.050*	−0.025
Income		0.019	0.008
Job title			
	(Professor vs. Assistant)	−0.085	−0.096
	(Associated professor vs. Assistant)	−0.147	−0.057
	(Lecturer vs. Assistant)	−0.140*	−0.066
Working hours		−0.038	−0.043
Number of SCI/SSCI		0.042	0.046
Turnover intention			
	(Never vs. frequently)	0.111	−0.127
	(Sometimes vs. frequently)	−0.067	−0.160*
Life events	(Yes vs. no)	−0.014	−0.023
Chronic disease	(Yes vs. no)	−0.238***	−0.180***
Organizational behavior variables			
group identification			0.121***
organizational justice			0.014
POS			0.055
psychological empowerment			0.075
PsyCap			0.335***
Adjusted R^2^		0.114***	0.333***
ΔR^2^		0.131	0.219

*POS* perceived organizational support; *PsyCap* psychological capital; *PCS* physical component summary

*p < 0.05, **p < 0.01, ***p < 0.001

**Table 4:** Hierarchical multiple regression predicting the MCS scores

Variable		MCS	
		Step1(β)	Step2(β)
Demographic and work characteristics			
Age		0.006	0.007
Gender	(Man vs. Women)	−0.007	−0.004
Marital status			
	(Single vs. Married/cohabitation)	−0.067	−0.069*
	(Divorced/others vs. Married/cohabitation)	0.059	0.034
Educational level			
	(Doctor vs. bachelor)	−0.098	−0.092*
	(master vs. bachelor)	−0.161**	−0.105*
Working years		−0.051*	−0.018*
Income		0.062	0.047
Job title			
	(Professor vs. Assistant)	−0.048	−0.062
	(Associated professor vs. Assistant)	−0.138	−0.027
	(Lecturer vs. Assistant)	−0.093	0.002
Working hours		−0.049	−0.051
Number of SCI/SSCI		−0.005	−0.002
Turnover intention			
	(Never vs. frequently)	0.261**	−0.032
	(Sometimes vs. frequently)	0.077	−0.035
Life events	(Yes vs. no)	−0.042	−0.050
Chronic disease	(Yes vs. no)	−0.188***	−0.112***
Organizational behavior variables			
group identification			0.107***
organizational justice			0.002
POS			0.124**
psychological empowerment			0.093*
PsyCap			0.420***
Adjusted R^2^		0.105***	0.455***
ΔR^2^		0.122	0.347

*POS* perceived organizational support; *PsyCap* psychological capital; *MCS* mental component summary

*p < 0.05, **p < 0.01, ***p < 0.001

## Discussion

The results described indicate that Chinese college teachers undergo a lower level of QOL both physically and mentally compared with Chinese general population [46, 47], which was consistent with previous studies [9, 10]. College teachers’ mental QOL was more seriously impaired than physical QOL. Organizational behavior factors (PsyCap, group identification, POS and psychological empowerment) are significant positive predictors of college teachers’ HRQOL.

### Demographic variables

In this study, demographic and work characteristics of college teachers explained 13.1 % variance of PCS and 12.2 % variance of MCS. Gender difference of PCS and MCS was not spotted. Among all the demographic and work characteristics, chronic disease was the strongest predictor of both PCS and MCS. This result is supported by previous findings that chronic disease is the largest threat to health status and it impacts people’s daily functioning and well-being [48–50]. Moreover, according to the WHO World Health Survey, people with chronic physical diseases are more likely to suffer from mental disorder like depression [51] which is also in support of our results. Teachers who are ≤30 years old had the highest level of PCS and MCS. This was reasonable since these college teachers were young and energetic, and the requirements from work like publishing papers and professional ranks and titles were not so urgent. However, in the regression analysis, age was not a predictor of physical and mental QOL. In the regression model, single teachers were more likely to have a lower level of mental QOL compare with those were married or cohabitation. This is in line with previous researches which showed that married individuals report lower level of mental distress and higher level of life satisfaction and subjective well-being [52].

### Work characteristics

As for educational level and job title, teachers with doctor and master degree tended to have impaired mental QOL in comparison with those with bachelor degree, while assistants reported better physical QOL than professors, associated professors and lecturers. This may be because college teachers with higher educational level and job title are often pace-setter in scientific research and bear more teaching and research tasks. So these teachers may experience much more pressure and stress during work, together with long time overcommitment to work and sleep deprivation, resulting in lower level of physical and mental QOL. Furthermore, those never have turnover intentions reported considerably higher PCS and MCS than those sometimes or frequently have. It can be explained that those who have turnover intention are likely to have burnout [53], reduced job satisfaction [54] and low quality of work life [55] which may cause adverse impact on college teachers’ QOL.

### Organizational behavior variables

The main finding of the present study is that organizational behavior factors are strong predictors of college teachers’ physical and mental QOL. Organizational behavior variables (PsyCap, group identification, organizational justice, POS and psychological empowerment) together contribute 21.9 % and 34.7 % to the variance of PCS and MCS respectively. In the hierarchical multiple regression analysis, group identification and PsyCap were significantly positively related to college teachers’ physical QOL and group identification, POS, psychological empowerment and PsyCap were significantly positively related to mental QOL. This is in accord with aforementioned studies which suggested that group identification [22, 23], POS [32–34], psychological empowerment [28] [37] and PsyCap [14, 18, 19] are positively associated with employees’ physical and/or mental health.

Among all the organizational behavior variables, PsyCap was the strongest predictor for both PCS and MCS, as the standardized coefficients of PsyCap in the regression model for PCS (β = 0.335) and MCS (β = 0.420) were both the largest among all the organizational behavior variables. This finding was reasonable since PsyCap is a higher-order core construct drawn from positive organizational behavior. Organizational factors like empowerment, organizational support and transformational leadership are effective on PsyCap [56]. Moreover, PsyCap may play the role of mediator between OB variables and their outcome variables under multi-factor circumstances. For example, Liu et al. reported that PsyCap significantly mediated the association between POS and depressive symptoms among Chinese male correctional officers [57]. This suggests that, for college managers, teachers’ PsyCap should be their first consideration and is the key point in the process of protecting and improving college teacher’ QOL from the perspective of organizational behavior. PsyCap is a kind of positive psychological state of an individual in the process of growth and development [14] and its four state-like psychological resource capacities can all be measured, developed and managed [58, 59]. So the enhancement of teachers’ PsyCap should be incorporated and attached importance to in the strategy for the protection and improvement of college teachers’ QOL both physically and mentally.

Meanwhile, group identification, psychological empowerment should also be the concern of school managers in protecting and improving teachers’ HRQOL. In this study, the positive effects of psychological empowerment on college teachers’ mental QOL and the positive effects of group identification on both physical and mental QOL were confirmed. Group identification is an individual’s subjective sense of belonging to a group and of commonality with other in-group members [60], and it provides one with a sense of meaning, permanence, and stability [22]. Previous researches had confirmed the positive effects of group identification on group commitment [61] and performance [62]. Similarly, higher perceived psychological empowerment is associated with employee’s increased job performance and job satisfaction [36, 63, 64], lower job strain [65], and lower job insecurity [66]. These heightened work behaviors, attitudes, outcomes and positive psychological feelings may in turn bring positive influence on teachers’ physical and mental condition, resulting in their better QOL. Moreover, POS also has positive effects on college teachers’ mental QOL. The stronger those teachers perceive that school managers value their contribution and are concerned with their well-being, the better college teachers’ mental QOL is. More specifically, the more teachers receive supervisor support, organizational rewards and favorable job conditions [67], the stronger their POS, and the better their mental QOL.

The implication of the abovementioned results for college managers is that interventions that focus on the PsyCap, group identification, POS and psychological empowerment of college teachers will contribute to better physical and mental QOL of teachers. For the sake of better HRQOL of college teachers and better quality of high education, relative measures should be taken to improve and strengthen teachers’ PsyCap, group identification, POS and psychological empowerment at work.

However, unexpectedly, in the hierarchical multiple regression models, organizational justice were not a significant predictor of college teachers’ physical and mental QOL, though it was positively correlated with both PCS and MCS in the correlation analysis. As abundant studies have confirmed the positive impacts of organizational justice on health [28, 68–70], one probable explanation is that, the effects of organizational justice on PCS and MCS were mediated by other studied organizational behavior variables. For example, previous organizational behavior researches have affirmed that organizational justice was predictor of group identification [71], POS [72] and psychological empowerment [73]; group identification [74] and POS [75–77] are mediators in the relation between organizational justice and various outcome variables.

It should be emphasized that the present study bears some limitations. Firstly, it is characterized by cross-sectional design which prevents us from making any causal statements about the association observed between organizational behavior variables and QOL. Whether changing these organizational behavior variables would improve teachers’ QOL needs further longitudinal research to confirm. However, in the data analysis, we found that “chronic disease” was significantly correlated with organizational behavior variables, and “working years” was a significant predictor for both PCS and MCS, which makes it more plausible that organizational factors may influence HRQOL. Secondly, common method variance may artificially inflate relationships between variables since all the data in our research were collected with self-reported measures. In further research, self-reported measures, organizational records and face-to-face interview will be employed to avoid this problem. Finally, all the participants were from colleges only in Shenyang city, which may limit the generalizability of this study to other populations.

## Conclusions

Chinese college teachers experienced relatively low level of HRQOL and their mental QOL were impaired more seriously than physical QOL. The main findings reported in our study suggest that organizational behavior factors (PsyCap, group identification, POS and psychological empowerment) were strong predictors for both of college teachers’ physical and mental QOL and are positive resources for improving teachers’ HRQOL. The enhancement of college teachers’ PsyCap, group identification, POS and psychological empowerment at work should be incorporated in the strategy of protecting and improving college teachers’ HRQOL.

## Abbreviations

QOL: Quality of Life
HRQOL: Health Related Quality of Life
SF-36: the 36-item Short-Form Health Survey
PCS: Physical Component Summary
MCS: Mental Component Summary
PsyCap: Psychological Capital
POS: Perceived Organizational Support
